# Association Between Body Roundness Index and Cancer Risk, With Further Stratification by Cardiometabolic Disease Status: Findings From Three National Longitudinal Cohorts

**DOI:** 10.1002/cam4.71324

**Published:** 2025-10-29

**Authors:** Pincheng Luo, Yanxue Lian

**Affiliations:** ^1^ School of Medicine University of Galway Galway Ireland

**Keywords:** body roundness index, cancer incidence, cardiometabolic disease, obesity

## Abstract

**Objective:**

This study investigates the association between body roundness index (BRI) and overall cancer incidence in a large population‐based cohort and explores the consistency of this association in individuals with and without cardiometabolic disease (CMD).

**Design:**

A prospective cohort study.

**Setting:**

Data for this study was extracted from three cohorts: the English Longitudinal Study of Aging, the Health and Retirement Study, and the China Health and Retirement Longitudinal Study.

**Participants:**

Among 33,624 individuals aged 50 and older, 2999 reported a cancer diagnosis.

**Measurements:**

BRI was calculated using measured height and waist circumference, while cancer and CMD were based on self‐reported physician diagnoses. Restricted cubic spline curves assessed nonlinearity and identified BRI cutoff points. Cox proportional hazards models were used to evaluate the association between BRI and cancer incidence, with subgroup and interaction analyses conducted by CMD status.

**Results:**

A non‐linear relationship was identified between BRI and cancer incidence, with an inflection point at BRI = 5.06. Participants with BRI > 5.06 had a 10% higher risk of cancer (HR: 1.10; 95% CI: 1.02–1.19) compared to those with BRI ≤ 5.06, while increases in BRI below this threshold were not significantly associated with risk. This remained consistent across CMD‐stratified analyses. No significant interaction was detected between BRI and CMD status.

**Conclusion:**

BRI is associated with cancer risk in a non‐linear manner, with increased risk above a defined threshold, irrespective of CMD status. These findings highlight the potential of BRI as a simple tool for cancer risk screening in older adults.

## 1 Introduction

Cancer remains one of the leading causes of morbidity and mortality worldwide. According to the World Health Organization, there were 20 million new cases and 9.7 million deaths in 2022 [1]. Approximately 40% of cancer cases are linked to modifiable lifestyle factors, while 4%–8% are specifically associated with excess body weight [2, 3, 4]. Obesity is commonly assessed using Body Mass Index (BMI), a widely used and easily calculated measure. However, BMI has notable limitations; it does not differentiate between fat and lean mass, nor does it account for fat distribution [5], which may lead to inaccurate risk assessment in individuals with varying body compositions. In contrast, the Body Roundness Index (BRI) has gained attention as a novel anthropometric measure that incorporates waist circumference and height to evaluate body shape and fat distribution, particularly central adiposity [6]. BRI is strongly correlated with visceral adiposity [6], which may contribute to cancer development through various biological mechanisms [7, 8]. While the association between BMI and cancer risk has been extensively studied, the relationship between BRI and overall cancer incidence remains largely unexplored. Investigating this association may provide new insights into the role of central adiposity in cancer development.

Additionally, cardiometabolic disease (CMD) frequently coexists with obesity and is closely interrelated. CMDs may contribute to systemic inflammation and other biological disturbances that promote cancer development [9, 10, 11, 12]. As the population ages, CMDs are becoming increasingly prevalent, with 4.7% of adults aged 60 and older affected by at least two of these conditions [13]. However, it remains unclear whether CMD modifies the association between BRI and cancer risk, and whether this association is consistent in individuals with and without CMD has not been systematically investigated.

Therefore, this study aims to investigate the association between BRI and overall cancer incidence in a large population‐based cohort. Furthermore, we sought to determine whether this association differs according to CMD status, thereby providing new insights into the complex interplay between adiposity, metabolic health, and cancer risk. These findings may contribute to a better understanding of obesity‐related cancer risk and inform future public health strategies aimed at cancer prevention in diverse metabolic populations.

## 2 Method

### 2.1 Study Design and Participants

Data for this study were extracted from three aging cohorts: the English Longitudinal Study of Aging (ELSA) [14], the Health and Retirement Study (HRS) [15], and the China Health and Retirement Longitudinal Study (CHARLS) [16]. These cohorts followed similar survey protocols with biennial (every 2 years) data collection, enabling cross‐regional comparisons and consistent follow‐up intervals for participants. We used data from waves 8–12 of the HRS, waves 2–6 of the ELSA (excluding waves 3 and 5 because they did not include waist circumference measurements necessary for calculating BRI), and waves 1–3 of the CHARLS to define the baseline. For each participant, the earliest wave within the specified ranges in which exposure data (i.e., BRI) were available was defined as their baseline wave. Baseline covariates were extracted from the same wave to ensure that exposure and covariate data were measured contemporaneously. Follow‐up extended to wave 15 in the HRS, wave 9 in the ELSA, and wave 5 in the CHARLS (Figure S1).

Participants with missing data on exposure or outcomes, as well as those who reported a cancer diagnosis before baseline, were excluded. Additionally, a small number of measurements were deemed implausible due to potential recording or measurement errors; therefore, individuals with BRI values in the < 1st or > 99th percentile were excluded to minimize data inconsistencies. Participants younger than 50 years or those missing key demographic information (age, gender, and marital status) were also excluded. After applying these criteria, the final analytical sample comprised 15,229 participants from CHARLS, 9287 from ELSA, and 14,374 from HRS (Figure S2). All studies received ethical approval from the respective local research ethics committees, and participants provided written informed consent before enrollment.

### 2.2 Measures and Outcomes

Standing height (in meters) and waist circumference (in centimeters) were recorded as part of the study measurements. The BRI was computed using the formula proposed by Thomas et al. [6]:

$$\begin{array}{c} \mathrm{BRI}=364.2-365.5\\ \;\times \sqrt{1-{\left(\frac{\text{waist circumference}\;\left(\mathrm{cm}\right)}{2\pi }\right)}^{2}÷{\left(50\times \text{height}\left(m\right)\right)}^{2}} \end{array}$$

Cancer status was assessed as a binary variable (“yes” or “no”), based on participants' self‐reported physician‐confirmed diagnoses. As the exact date of diagnosis was not collected, for individuals who reported having cancer, the estimated time of diagnosis was determined as the midpoint between the time of the wave in which cancer was first reported and the time of the last wave in which no diagnosis was indicated. Follow‐up time was calculated from this estimated diagnosis time to baseline. For participants without a cancer diagnosis, follow‐up duration was defined as the period from the time of their last recorded study wave to baseline.

### 2.3 Covariates

Socioeconomic status was determined based on education level and country‐specific household wealth quartiles [17]. Demographic factors included age (categorized as 50–74 or 75+), gender (male or female), and marital status (married/partnered or unmarried/other). Country was also included as an adjustment variable. Lifestyle factors comprised smoking status (never, former, or current), alcohol consumption (yes or no), and physical activity level (active or inactive). While CMDs encompass various conditions, this study specifically considered diabetes, heart disease, and stroke, as defined in earlier research [9]. Hypertension was analyzed separately as a major risk factor for chronic conditions.

### 2.4 Statistical Analysis

A restricted cubic spline (RCS) model with three knots placed at the 10th, 50th, and 90th percentiles of the BRI distribution was employed to flexibly assess the potential non‐linear relationship between BRI and overall cancer incidence. The number and placement of knots were selected based on commonly used statistical methods [18, 19]. The optimal cutoff point for BRI was derived from the turning point on the spline curve where the hazard ratio stabilized, based on the first derivative of the fitted model. If a non‐linear association was detected, BRI was categorized accordingly based on the determined cutoffs. Stepwise‐adjusted Cox proportional hazards models were used to examine the relationship between BRI and cancer incidence. Model I was unadjusted, while Model II accounted for age, gender, marital status, socioeconomic status, and country. Model III, the fully adjusted model, further incorporated lifestyle factors (smoking, alcohol consumption, and physical activity) and chronic diseases, including hypertension and CMD.

Additionally, to evaluate whether the BRI–cancer association varied by CMD status, we conducted separate RCS and Cox regression analyses within subgroups of individuals with and without CMD. This allowed us to explore potential non‐linear associations and assess their consistency with the overall population findings. To formally test for effect modification, an interaction term between BRI and CMD status was included in the multivariable model, and the *p*‐value for interaction was obtained using the Wald test.

Multiple imputation by chained equations was used to address missing data on covariates. To assess the robustness of our findings, we conducted three sensitivity analyses. First, we repeated the analysis using only complete cases. Second, to minimize potential reverse causation, we excluded participants with < 2 years of follow‐up and reanalyzed the data. Third, given the distinct ethnic backgrounds of participants from the three countries included in the study, we conducted separate analyses within each national cohort and then pooled the results using meta‐analysis techniques. In addition, stratified analyses were performed by age, sex, socioeconomic status, and marital status to explore the relationship between BRI and cancer incidence across different subgroups. Results were reported as hazard ratios (HRs) with 95% confidence intervals (CIs), and statistical significance was set at *p* < 0.05. All analyses were performed using Stata 18.0.

## 3 Result

Among 33,624 individuals aged 50 and older with baseline measurements and cancer information, the mean age was 62.39 [standard deviation (SD): 8.63] years, and 9640 had self‐reported CMDs (Table 1). Over a mean follow‐up of 9.01 (3.16) years, 2999 participants reported a cancer diagnosis. Their mean BRI was 5.50 (1.85), which was higher than that of individuals without cancer. Additionally, those diagnosed with cancer were more likely to be older, female, physically inactive, have a history of smoking or be current smokers, and consume alcohol. To assess potential selection bias due to missing exposure, outcome data, key baseline variables, or extreme values, we compared baseline characteristics between included and excluded participants. Substantial differences were observed (Table S1), which should be considered when interpreting the findings.

**TABLE 1:** Baseline characteristics of the study population by cancer status.

	Total	Non‐cancer	Cancer	*p*
***N* (%)**	33,624 (100)	30,625 (91.08)	2999 (8.92)	
**BRI, mean (SD)**	5.06 (1.80)	5.02 (1.79)	5.50 (1.85)	< 0.001
**Age, years**	62.39 (8.63)	62.08 (8.54)	65.57 (8.96)	< 0.001
50–74	30,008	27,514 (91.69)	2494 (8.31)	
75+	3616	3111 (86.03)	505 (13.97)	
**Gender, *N* (%)**				< 0.001
Male	15,255	13,764 (90.23)	1491 (9.77)	
Female	18,369	16,861 (91.79)	1508 (8.21)	
**Marital status, *N* (%)**				< 0.001
Married or partnered	24,140	22,071 (91.43)	2069 (8.57)	
Unmarried or others	9484	8554 (90.19)	930 (9.81)	
**Socioeconomic status, *N* (%)**				< 0.001
Low	12,431	11,626 (93.52)	805 (6.48)	
Middle	14,233	13,004 (91.37)	1229 (8.63)	
High	6960	5995 (86.14)	965 (13.86)	
**Smoking, *N* (%)**				< 0.001
Never	16,267	15,126 (92.99)	1141 (7.01)	
Former	10,402	9045 (86.95)	1357 (13.05)	
Current	6955	6454 (92.80)	501 (7.20)	
**Drinking, *N* (%)**				< 0.001
Yes	17,852	16,064 (92.32)	1788 (7.68)	
No	15,772	14,561 (89.98)	1211 (10.02)	
**Physical activity, *N* (%)**				0.195
Active	10,379	9422 (90.78)	957 (9.22)	
Inactive	23,245	21,203 (91.22)	2042 (8.78)	
**Hypertension, *N* (%)**				< 0.001
Yes	18,590	16,652 (89.58)	1938 (10.42)	
No	15,034	13,973 (92.94)	1061 (7.06)	
**CMD, *N* (%)**				< 0.001
Yes	9640	8420 (87.34)	1220 (12.66)	
No	23,984	22,205 (92.58)	1779 (7.42)	
**Follow‐up, years, mean (SD)**	9.01 (3.16)	9.37 (2.87)	5.35 (3.62)	< 0.001
**Country/Region, *N* (%)**				< 0.001
China	11,287	11,181 (99.06)	106 (0.94)	
England (UK)	9136	8173 (89.46)	963 (10.54)	
US	13,201	11,271 (85.38)	1930 (14.62)	

*Note:* Continuous variables are expressed as mean (SD), while categorical variables are presented as frequency (percentage).

Abbreviations: BRI, body roundness index; CMD, cardiometabolic disease; SD, standard deviation.

**FIGURE 1:**
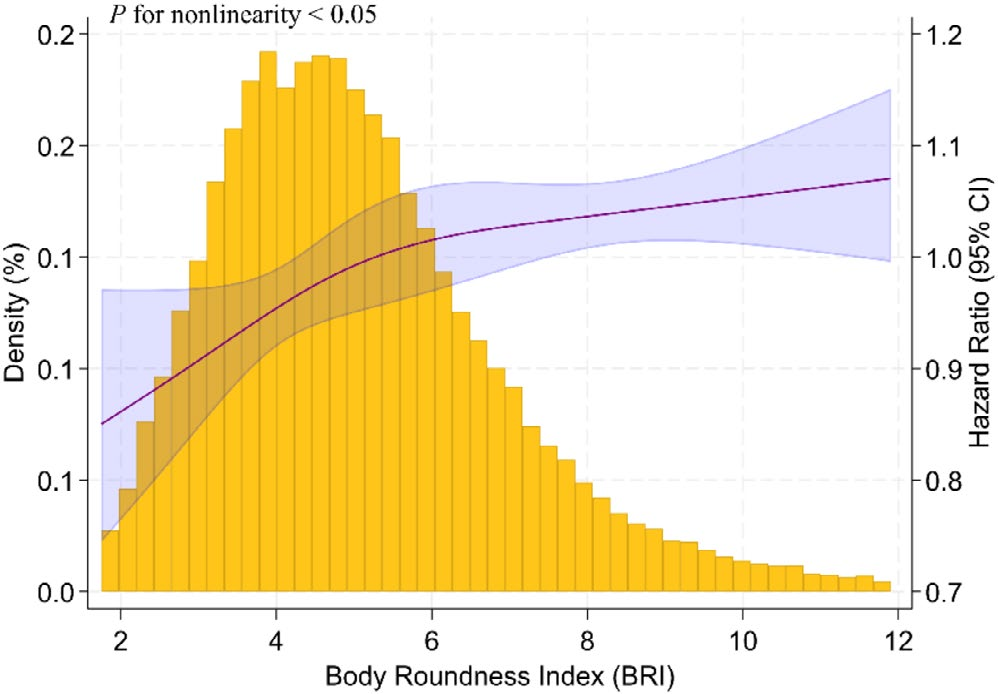
RCS Curve of the association between BRI and cancer incidence after full adjustment.

An RCS curve was first employed to assess the association between the BRI and overall cancer incidence (Figure 1), given the absence of established cutoff points for BRI. The analysis revealed a non‐linear trajectory resembling a reversed hockey stick, with an inflection point at BRI = 5.06. Based on this threshold, participants were categorized into two groups. Those with BRI > 5.06 were more likely to be older and to have hypertension, CMD, and cancer (Table S2), while individuals with BRI ≤ 5.06 were designated as the reference group. The cancer incidence rate was higher among those with elevated BRI (12.11 per 1000 person‐years) compared to those with lower BRI (8.14 per 1000 person‐years). Correspondingly, the higher BRI group demonstrated a significantly increased cancer risk, with a fully adjusted hazard ratio of 1.10 (95% CI, 1.02–1.19), indicating a 10% greater likelihood of developing cancer (Table 2).

**TABLE 2:** Adjusted HR of cancer incidence according to the categories of BRI.

BRI classification	Person‐year	Incidence rate (per 1000 person‐years)	Model I	Model II	Model III
**Binary classification based on BRI cutoff (≤ 5.06 vs > 5.06)**					
≤ 5.06	168,579.3	8.14	Reference	Reference	Reference
> 5.06	134,295.3	12.11	1.47 (1.37; 1.58)	1.12 (1.03; 1.20)	1.10 (1.02; 1.19)
**Classification based on BRI cutoff (5.06) and within‐group medians (Groups 1–4)**					
Group 1 (1.74–3.87)	83,690	6.76	Reference	Reference	Reference
Group 2 (3.88–5.06)	84,889.33	9.51	1.39 (1.25; 1.55)	1.10 (0.97; 1.23)	1.10 (0.98; 1.24)
Group 3 (5.07–6.26)	66,837.83	11.12	1.62 (1.45; 1.81)	1.16 (1.03; 1.31)	1.15 (1.02; 1.30)
Group 4 (6.27–11.91)	67,457.5	13.09	1.90 (1.71; 2.11)	1.21 (1.07; 1.36)	1.18 (1.05; 1.34)

*Note:* Model I, unadjusted model. Model II, adjusted for age, gender, marital status, socioeconomic status, and country. Model III, further adjusted for lifestyle factors and chronic diseases, including smoking, drinking, physical activity, hypertension, and cardiometabolic disease.

Abbreviations: BRI, body roundness index; CMD, cardiometabolic disease.

Subsequently, participants were further divided into four groups (Groups 1–4) using the median BRI value within each of the two main categories (≤ 5.06 and > 5.06) to facilitate a more detailed risk assessment across BRI levels and to explore potential gradients in cancer risk. Compared to Group 1, cancer risk in Group 2 did not differ significantly (HR, 1.10; 95% CI, 0.98–1.24). However, a significant increase in cancer risk was observed in both Group 3 and Group 4, where BRI was > 5.06, further supporting the association between elevated BRI and increased cancer risk. Specifically, individuals in Group 3 had a 15% higher risk of cancer (HR, 1.15; 95% CI, 1.02–1.30), while those in Group 4 had an 18% higher risk (HR, 1.18; 95% CI, 1.05–1.34) compared to Group 1.

RCS analyses in both CMD subgroups revealed a similar non‐linear association between BRI and cancer risk, consistent with the overall population findings (Figure S3). Among individuals without CMD, a BRI above 5.06 was associated with a 7% higher cancer risk (HR: 1.07; 95% CI: 1.01–1.19) compared to those with a lower BRI. In individuals with CMD, the increase was more pronounced, with a 14% higher risk (HR: 1.14; 95% CI: 1.01–1.28) (Table 3). Moreover, interaction testing indicated no statistically significant interaction between BRI and CMD status (*p* for interaction > 0.05).

**TABLE 3:** Association between BRI and cancer incidence, stratified by CMD status.

BRI classification	Person‐year	Incidence rate (per 1000 person‐years)	Model I	Model II	Model III
**Without CMD**					
≤ 5.06	132733.2	7.12	Reference	Reference	Reference
> 5.06	82626.33	10.09	1.40 (1.27; 1.53)	1.12 (1.02; 1.23)	1.07 (1.01; 1.19)
**With CMD**					
≤ 5.06	35846.17	11.94	Reference	Reference	Reference
> 5.06	51,669	14.11	1.28 (1.14; 1.44)	1.13 (1.00; 1.28)	1.14 (1.01; 1.28)
***p* for interaction** ^a^			0.23	0.65	0.68

*Note:* Model I, unadjusted model. Model II, adjusted for age, gender, marital status, socioeconomic status, and country. Model III, further adjusted for lifestyle factors and chronic diseases, including smoking, drinking, physical activity, and hypertension.

Abbreviations: BRI, body roundness index; CMD, cardiometabolic disease.

^a^ *p* for interaction derived from the Wald test.

Results from all three sensitivity analyses were consistent with the primary analysis, reinforcing the robustness of the observed associations. When participants with a follow‐up duration of < 2 years were excluded, the association remained significant, with individuals having a BRI > 5.06 showing a higher risk of cancer (HR: 1.13; 95% CI: 1.03–1.24) (Table S3). Similarly, in a complete‐case analysis limited to participants with no missing covariate data, the association persisted and was stronger (HR: 1.49; 95% CI: 1.29–1.73) (Table S4). Additionally, a meta‐analysis synthesizing results across the three cohorts showed that individuals with a BRI > 5.06 had a 12% increased risk of cancer incidence compared to those with a BRI ≤ 5.06 (HR: 1.12; 95% CI: 1.04–1.21) (Figure S4). In stratified analyses, a BRI > 5.06 was associated with significantly elevated cancer risk among adults aged 50–74 years, individuals with low socioeconomic status, and those who were married or partnered, compared to their counterparts with BRI ≤ 5.06. No significant associations were observed in other subgroups (Figure S5).

Furthermore, we developed a visual reference for estimating cancer risk based on BRI, which is calculated from height and waist circumference, offering potential clinical guidance. Individuals with BRI < 5.06 (blue) generally exhibited lower cancer risk, whereas those with BRI ≥ 5.06 (orange) showed higher cancer risk (Figure S6). Notably, individuals with similar BRI values can have different combinations of height and waist circumference, underscoring the utility of BRI as a composite index. In particular, those with shorter stature but higher waist circumference appeared to be at greater cancer risk, highlighting this subgroup as a potential priority for targeted prevention efforts.

## 4 Discussion

This study is the first cross‐cultural and longitudinal investigation of the relationship between body shape, assessed via the BRI, and overall cancer incidence in older adults. We identified a non‐linear association with a clear threshold at a BRI of 5.06: cancer risk increased significantly above this value, whereas lower BRI levels were not substantially associated with risk. This threshold effect persisted across subgroups stratified by CMD status, with similar risk patterns observed in individuals with and without CMD. Importantly, no significant interaction was found between BRI and CMD status, indicating that CMD did not modify the BRI–cancer relationship. The robustness of this threshold effect was further supported by similar non‐linear trends in the RCS analyses across all groups.

Our findings are consistent with previous studies reporting an association between abdominal fat accumulation and cancer risk. A prospective study conducted in Denmark used whole‐body dual‐energy X‐ray absorptiometry to assess body fat distribution and examined the relationship between central obesity and overall cancer incidence in postmenopausal women [20]. The results showed that women with high central obesity had a 50% increased risk of cancer. Similarly, a study conducted in the United States found that each 1‐SD increase in visceral adipose tissue, as measured by computed tomography, was associated with a 43% higher risk of cancer [21].

In addition to these relatively expensive and less accessible imaging techniques, simpler anthropometric measures, such as waist‐to‐hip ratio and waist circumference, have also been used to estimate cancer risk. Individuals with higher waist‐to‐hip ratios and larger waist circumferences have been shown to have an elevated cancer risk [22, 23]. However, these indicators do not account for height, which may impact the accuracy of their reflection of central obesity, particularly for individuals who are exceptionally short or tall [5, 24]. To address this limitation, the BRI was developed. It incorporates both height and waist circumference by modeling the human body as an ellipse, with height representing the major axis and waist circumference the minor axis. Its value is calculated based on the ellipse's eccentricity, thereby providing a more precise estimation of body roundness and abdominal fat accumulation [25].

Excess body fat, particularly visceral fat surrounding internal organs, has been implicated in increased cancer risk through several interrelated biological pathways. Visceral adipose tissue is metabolically active and secretes pro‐inflammatory cytokines, fostering a state of chronic low‐grade inflammation that can promote DNA damage and initiate tumorigenesis [26]. Moreover, adipose tissue influences hormonal balance by elevating estrogen levels, particularly in postmenopausal women, thereby increasing the risk of hormone‐dependent cancers such as breast, endometrial, and ovarian cancers [27]. Obesity is also strongly associated with insulin resistance and compensatory hyperinsulinemia, both of which stimulate cell proliferation and inhibit apoptosis, processes that contribute to the development of malignancies such as colorectal and pancreatic cancer [26]. In addition, obesity alters adipokine profiles, with increased leptin and decreased adiponectin levels, creating a microenvironment that favors tumor growth [28]. Finally, obesity may impair immune surveillance, diminishing the body's ability to recognize and eliminate emerging cancer cells [29].

Although visceral fat is closely linked to the development of CMD, current evidence suggests that its pro‐carcinogenic effects may occur even in the absence of overt metabolic abnormalities [30]. Our findings are consistent with this perspective: the consistent association between BRI and cancer risk across individuals with and without CMD may reflect the independent carcinogenic potential of visceral adiposity. That is, excess visceral fat may promote cancer development through biological pathways that are not entirely dependent on the presence of clinically diagnosed CMD [31]. These mechanisms, including chronic inflammation, hormonal dysregulation, and immune impairment, may already be active in individuals with elevated BRI even in the absence of CMD, thereby contributing to cancer risk.

From a public health perspective, incorporating BRI into routine clinical and community assessments offers a simple, non‐invasive method for identifying individuals at elevated cancer risk, regardless of cardiometabolic disease status. Building on this, targeted interventions to maintain BRI below the identified threshold, through lifestyle changes such as improved diet, physical activity, and weight management, may contribute to cancer prevention, particularly in aging populations. Given the growing burden of cancer among older adults and the limitations of traditional obesity measures such as BMI, our findings highlight the potential value of BRI as a complementary tool in cancer risk stratification. Future interventional studies are warranted to validate these findings across diverse populations and to explore whether BRI‐guided prevention strategies can effectively reduce cancer incidence at the population level.

This study has several strengths. It utilized three large, nationally representative datasets and benefited from a relatively long follow‐up period (mean: 9.01 years), enhancing the reliability of the findings. Additionally, by employing a longitudinal measure of cancer incidence, the study captures clinically and biologically relevant changes over time. Stratified analyses based on the presence or absence of CMD further support the generalizability of the results across different health conditions. Multiple sensitivity analyses were also conducted, demonstrating the robustness and stability of the observed associations.

However, some limitations should be acknowledged. First, the markedly lower cancer incidence observed in the CHARLS cohort (0.94%) compared to ELSA (10.54%) and HRS (14.62%) may reflect underdiagnosis or underreporting in the Chinese population. This discrepancy could be due to differences in healthcare access, diagnostic capacity, and the accuracy of self‐reported data [16]. In addition to these systemic factors, cultural attitudes and stigma surrounding cancer in China may also lead to underreporting [32], potentially leading to misclassification and bias in incidence estimates. Second, as with other observational studies, our design limits the ability to establish a causal relationship between obesity, measured by BRI, and cancer incidence. Third, due to data collection methods, cancer diagnosis timing was estimated using wave midpoints, which may introduce measurement error and survival bias. Additionally, reliance on self‐reported outcomes may have led to underestimation from recall bias. Incomplete or inaccessible cancer subtype data, along with sample size limitations, restricted our analysis to overall cancer incidence. Fourth, our study excluded participants with missing exposure data, key baseline variables, outcome data, or extreme BRI values to ensure data validity. However, baseline comparisons revealed substantial differences between included and excluded individuals, indicating potential selection bias. As multiple imputations were not feasible for missing exposure or outcome data, this may affect the generalizability of our findings and should be considered in interpretation. Finally, although covariates were selected based on prior literature, residual confounding cannot be ruled out. In particular, dietary data, which can influence both body composition and cancer risk, were not available across the three cohorts we analyzed.

## 5 Conclusion

This study provides novel evidence of a non‐linear association between the BRI and overall cancer incidence in older adults, with a threshold effect identified at a BRI value of 5.06. Importantly, this association was consistent across individuals with and without CMD, and no significant interaction was observed between BRI and CMD status. These findings suggest that BRI may serve as a robust, easily obtainable tool for cancer risk stratification, independent of CMD status. The BRI should be considered for integration into public health practice, particularly for early screening and risk stratification in aging populations, following validation of these findings across more diverse cohorts and settings.

## Author Contributions

**Pincheng Luo:** conceptualization, investigation, methodology, validation, visualization, writing – review and editing, writing – original draft, formal analysis, data curation, software. **Yanxue Lian:** conceptualization, investigation, writing – original draft, methodology, validation, visualization, formal analysis, resources, supervision, data curation, software.

## Disclosure

We have not used any AI in the writing process.

## Ethics Statement

Ethical approval was obtained from local research ethics committees, and written informed consent was provided by all participants. These surveys were conducted in accordance with the ethical standards set forth in the 1964 Declaration of Helsinki and its subsequent amendments.

## Conflicts of Interest

The authors declare no conflicts of interest.

## Supporting information


**Table S1:** Baseline characteristics of included and excluded participants (exclusions due to missing key data or outlier exposure values).
**Table S2:** Baseline characteristics of the study population stratified by the identified BRI threshold of 5.06.
**Table S3:** Sensitive analysis after excluding those with follow‐up less than 2 years (< 24 months).
**Table S4:** Sensitivity analysis restricted to complete cases, excluding all observations with missing covariate data.
**Figure S1:** Study design.
**Figure S2:** Flowchart of data screening from the three cohort studies.
**Figure S3:** Fully adjusted restricted cubic spline curves showing the association between BRI and cancer incidence, stratified by cardiometabolic disease status.
**Figure S4:** Sensitivity analysis synthesizing results from three cohorts via meta‐analysis.
**Figure S5:** The association between BRI and cancer incidence across stratified subgroups.
**Figure S6:** BRI categories based on the inflection point and the distribution of height and waist circumferences among individuals aged 50 years and older.

## Data Availability

The data that support the finding is available from GATEWAY TO GLOBAL AGING DATA (https://g2aging.org/) [33].
