# Supplementary material for: Association Between Body Roundness Index and Cancer Risk, With Further Stratification by Cardiometabolic Disease Status: Findings From Three National Longitudinal Cohorts
Source: Cancer Med. 2025 Oct 29;14(21):e71324. doi: 10.1002/cam4.71324 (PMC12571965; doi:10.1002/cam4.71324)
Supplement: Supplementary file 1 — Table S1: Baseline characteristics of included and excluded participants (exclusions due to missing key data or outlier exposure values). Table S2: Baseline characteristics of the study population stratified by the identified BRI threshold of 5.06. Table S3: Sensitive analysis after excluding those with follow‐up less than 2 years (< 24 months). Table S4: Sensitivity analysis restricted to complete cases, excluding all observations with missing covariate data. Figure S1: Study design. Figure S2: Flowchart of data screening from the three cohort studies. Figure S3: Fully adjusted restricted cubic spline curves showing the association between BRI and cancer incidence, stratified by cardiometabolic disease status. Figure S4: Sensitivity analysis synthesizing results from three cohorts via meta‐analysis. Figure S5: The association between BRI and cancer incidence across stratified subgroups. Figure S6: BRI categories based on the inflection point and the distribution of height and waist circumferences among individuals aged 50 years and older. [file CAM4-14-e71324-s001.docx]

**Supplementary material**

**Table S1.** Baseline characteristics of included and excluded participants (exclusions due to missing key data or outlier exposure values).

**Table S2.** Baseline characteristics of the study population stratified by the identified BRI threshold of 5.06.

**Table S3.** Sensitive analysis after excluding those with follow-up less than 2 years (< 24 months).

**Table S4.** Sensitivity analysis restricted to complete cases, excluding all observations with missing covariate data.

**Figure S1.** Study design.

**Figure S2.** Flowchart of data screening from the three cohort studies.

**Figure S3.** Fully adjusted restricted cubic spline curves showing the association between BRI and cancer incidence, stratified by cardiometabolic disease status.

**Figure S4.** Sensitivity analysis synthesizing results from three cohorts via meta-analysis.

**Figure S5.** The association between BRI and cancer incidence across stratified subgroups.

**Figure S6**. BRI categories based on the inflection point and the distribution of height and waist circumferences among individuals aged 50 years and older.

**Table S1.** Baseline characteristics of included and excluded participants (exclusions due to missing key data or outlier exposure values).

|  | **Included** | **Excluded *** | ***P*-value** |
| --- | --- | --- | --- |
| **N** | 33624 | 14791 |  |
| **Age, years, Mean±SD** | 62.39±8.63 | 59.86±13.69 | <0.001 |
| **Gender, %** |  |  | 0.103 |
| Male | 45.37 | 46.17 |  |
| Female | 69.76 | 30.24 |  |
| **Marital status, %** |  |  | <0.001 |
| Married or partnered | 70.50 | 29.50 |  |
| Unmarried or others | 67.23 | 32.77 |  |
| **Socioeconomic status, %** |  |  | <0.001 |
| Low | 70.43 | 29.57 |  |
| Middle | 72.55 | 27.45 |  |
| High | 77.19 | 22.81 |  |
| **Smoking, %** |  |  | <0.001 |
| Never | 69.09 | 30.91 |  |
| Former | 74.04 | 25.96 |  |
| Current | 69.08 | 30.92 |  |
| **Drinking, %** |  |  | <0.001 |
| Yes | 74.57 | 25.43 |  |
| No | 67.79 | 32.21 |  |
| **Physical activity, %** |  |  | <0.001 |
| Active | 70.08 | 29.92 |  |
| Inactive | 78.88 | 21.12 |  |
| **Hypertension, %** |  |  | <0.001 |
| Yes | 76.48 | 23.52 |  |
| No | 96.55 | 3.45 |  |
| **CMD, %** |  |  | <0.001 |
| Yes | 72.74 | 27.26 |  |
| No | 78.23 | 21.77 |  |
| **Country/ Region, %** |  |  | <0.001 |
| China | 62.05 | 37.95 |  |
| England (UK) | 74.45 | 25.55 |  |
| U.S. | 73.53 | 26.47 |  |

***** Excluded participants include those removed due to missing exposure (BRI measurements) or outcome data (cancer status), missing key baseline variables (age, gender, or marital status), or extreme values of the exposure (e.g., <1st percentile or >99th percentile). **Abbreviation:** SD, standard deviation; CMD, cardiometabolic disease.

**Table S2.** Baseline characteristics of the study population stratified by the identified BRI threshold of 5.06.

|  | **Total** | **<= 5.06** | **> 5.06** | ***P*-value** |
| --- | --- | --- | --- | --- |
| **N** | 33,624 (100) | 18,822 (55.98) | 14,802 (44.02) |  |
| **BRI, mean (SD)** | 5.06 (1.80) | 3.80 (0.80) | 6.67 (1.41) | <0.001 |
| **Age, years, mean (SD)** | 62.39 (8.63) | 61.70 (8.37) | 63.28 (8.88) | <0.001 |
| 50-74 | 30,008 | 17,111 (57.02) | 12,897 (42.98) |  |
| 75+ | 3,616 | 1,711 (47.32) | 1,905 (52.68) |  |
| **Gender, N (%)** |  |  |  | <0.001 |
| Male | 15,255 | 9,481 (51.61) | 8,888 (48.39) |  |
| Female | 18,369 | 9,341 (61.23) | 5,914 (38.77) |  |
| **Marital status, N (%)** |  |  |  | <0.001 |
| Married and partnered | 24,140 | 14,098 (58.40) | 10,042 (41.60) |  |
| Unmarried and others | 9,484 | 4,724 (49.81) | 4,760 (50.19) |  |
| **Socioeconomic status, N (%)** |  |  |  | <0.001 |
| Low | 12,431 | 6,709 (53.97) | 5,722 (46.03) |  |
| Middle | 14,233 | 7,858 (55.21) | 6,375 (44.79) |  |
| High | 6,960 | 4,255 (61.14) | 2,705 (38.86) |  |
| **Smoking, N (%)** |  |  |  | <0.001 |
| Never | 16,267 | 9,025 (55.48) | 7,242 (44.52) |  |
| Former | 10,402 | 4,960 (47.68) | 5,442 (52.32) |  |
| Current | 6,955 | 4,837 (69.55) | 2,118 (30.45) |  |
| **Drinking, N (%)** |  |  |  | <0.001 |
| Yes | 17,852 | 10,406 (58.29) | 7,446 (41.71) |  |
| No | 15,772 | 8,416 (53.36) | 7,356 (46.64) |  |
| **Physical activity, N (%)** |  |  |  | <0.001 |
| Active | 10,379 | 6,598 (63.57) | 3,781 (36.43) |  |
| Inactive | 23,245 | 12,224 (52.59) | 11,021 (47.41) |  |
| **Hypertension, N (%)** |  |  |  | <0.001 |
| Yes | 18,590 | 8,403 (45.20) | 10,187 (54.80) |  |
| No | 15,034 | 10,419 (69.30) | 4,615 (30.70) |  |
| **CMD, N (%)** |  |  |  | <0.001 |
| Yes | 9,640 | 3,965 (41.13) | 5,675 (58.87) |  |
| No | 23,984 | 14,857 (61.95) | 9,127 (38.05) |  |
| **Cancer, N (%)** |  |  |  | <0.001 |
| Yes | 2,999 | 1,373 (45.78) | 1,626 (54.22) |  |
| No | 30,625 | 17,449 (56.98) | 13,176 (43.02) |  |
| **Follow-up, years** | 9.01 (3.16) | 8.96 (3.02) | 9.07 (3.33) | <0.001 |
| **Country/ Region, N (%)** |  |  |  |  |
| China | 11,287 | 8,152 (72.22) | 3,135 (27.78) |  |
| England (UK) | 9,136 | 5,118 (56.02) | 4,018 (43.98) |  |
| U.S. | 13,201 | 5,552 (42.06) | 7,649 (57.94) |  |

Continuous variables are expressed as mean (standard deviation), while categorical variables are presented as frequency (percentage). **Abbreviation:** SD, standard deviation; BRI, body roundness index; CMD, cardiometabolic disease.

**Table S3.** Sensitive analysis after excluding those with follow-up less than 2 years (< 24 months).

| **BRI classification** | **Person-year** | **Incidence Rate (****per 1,000 person-years)** | **Model I** | **Model II** | **Model III** |
| --- | --- | --- | --- | --- | --- |
| **Binary classification based on BRI cutoff (****≤5.06 vs >5.06)** | | | | | |
| <= 5.06 | 168185.9 | 6.31 | Reference | Reference | Reference |
| > 5.06 | 133839.9 | 9.34 | 1.45 (1.34; 1.57) | 1.14 (1.05; 1.25) | 1.13 (1.03; 1.24) |
| **Classification based on BRI cutoff (5.06) and within-group medians (Groups 1–4)** | | | | | |
| Group 1 (1.74-3.87) | 83528.75 | 5.23 | Reference | Reference | Reference |
| Group 2 (3.88-5.06) | 84657.17 | 7.37 | 1.38 (1.22; 1.56) | 1.10 (0.96; 1.25) | 1.10 (0.97; 1.26) |
| Group 3 (5.07-6.26) | 66621.92 | 8.47 | 1.58 (1.39; 1.79) | 1.17 (1.02; 1.33) | 1.16 (1.01; 1.33) |
| Group 4 (6.27,11.91) | 67218 | 10.21 | 1.88 (1.67; 2.12) | 1.25 (1.09; 1.43) | 1.24 (1.08; 1.43) |

Model I, unadjusted model. Model II, adjusted for age, gender, marital status, socioeconomic status, and country. Model III, further adjusted for lifestyle factors and chronic diseases, including smoking, drinking, physical activity, hypertension, and cardiometabolic diseases. **Abbreviation:** BRI, body roundness index.

**Table S4.** Sensitivity analysis restricted to complete cases, excluding all observations with missing covariate data.

| **BRI classification** | **Person-year** | **Incidence Rate (per 1,000 person-years)** | **Model I** | **Model II** | **Model III** |
| --- | --- | --- | --- | --- | --- |
| **Binary classification based on BRI cutoff (≤5.06 vs >5.06)** | | | | | |
| <= 5.06 | 54866.42 | 6.27 | Reference | Reference | Reference |
| > 5.06 | 53058.25 | 11.44 | 1.43 (1.29; 1.59) | 1.19 (1.06; 1.32) | 1.49 (1.29; 1.73) |
| **Classification based on BRI cutoff (5.06) and within-group medians (Groups 1–4)** | | | | | |
| Group 1 (1.74-3.87) | 26,441.17 | 5.52 | Reference | Reference | Reference |
| Group 2 (3.88-5.06) | 28,425.25 | 6.97 | 1.22 (1.04; 1.42) | 1.07 (0.91; 1.25) | 1.15 (0.92; 1.43) |
| Group 3 (5.07-6.26) | 23,581.50 | 9.88 | 1.46 (1.25; 1.71) | 1.18 (1.01; 1.39) | 1.49 (1.19; 1.86) |
| Group 4 (6.27,11.91) | 29476.75 | 12.69 | 1.73 (1.49; 2.01) | 1.28 (1.09; 1.50) | 1.75 (1.41; 2.17) |

Model I, unadjusted model. Model II, adjusted for age, gender, marital status, socioeconomic status, and country. Model III, further adjusted for lifestyle factors and chronic diseases, including smoking, drinking, physical activity, hypertension, and cardiometabolic diseases. **Abbreviation:** BRI, body roundness index.


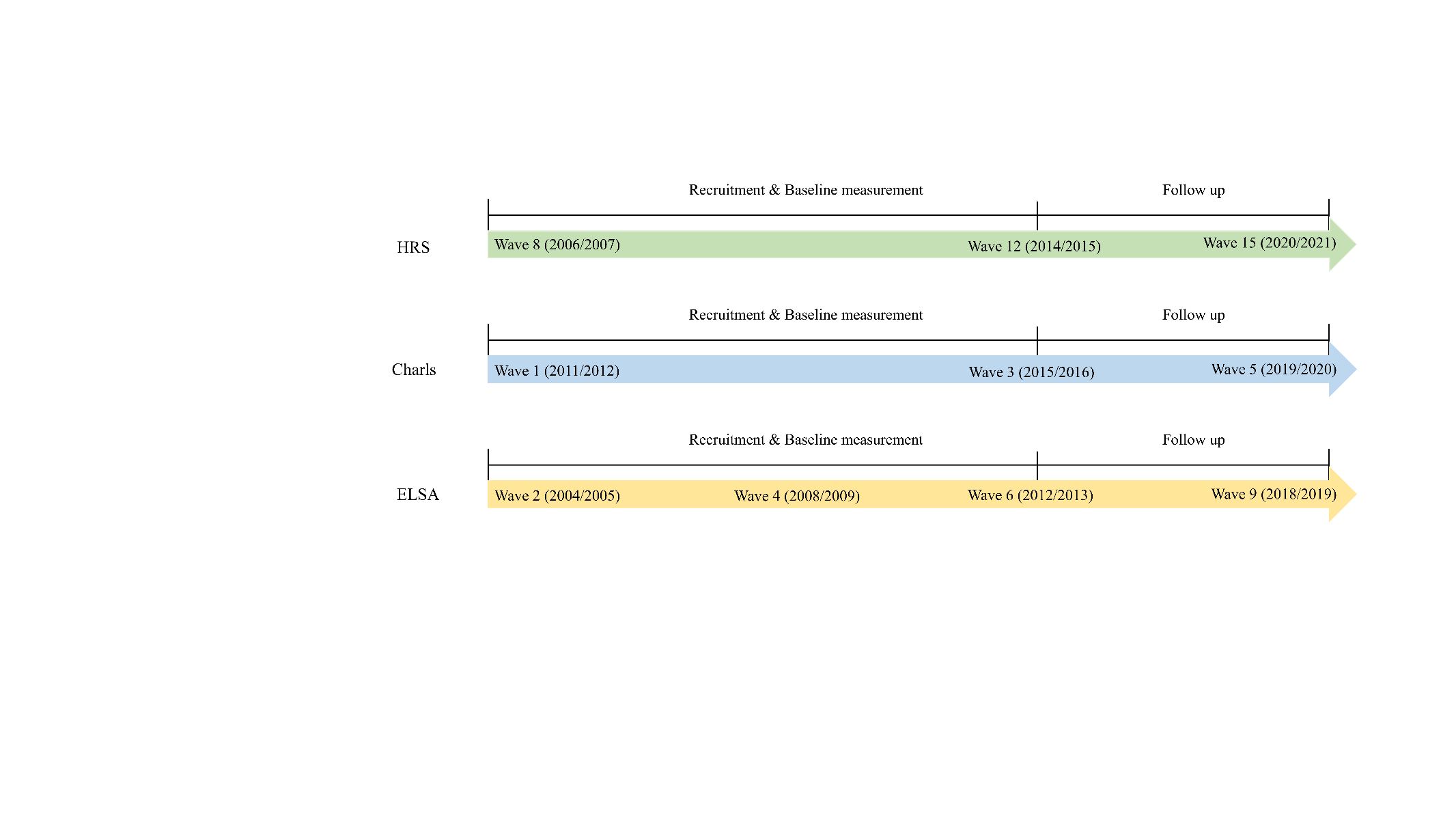


**Figure S1.** Study design. **Abbreviation:** ELSA, the English Longitudinal Study of Ageing; HRS, the Health and Retirement Study; CHARLS, the China Health and Retirement Longitudinal Study.


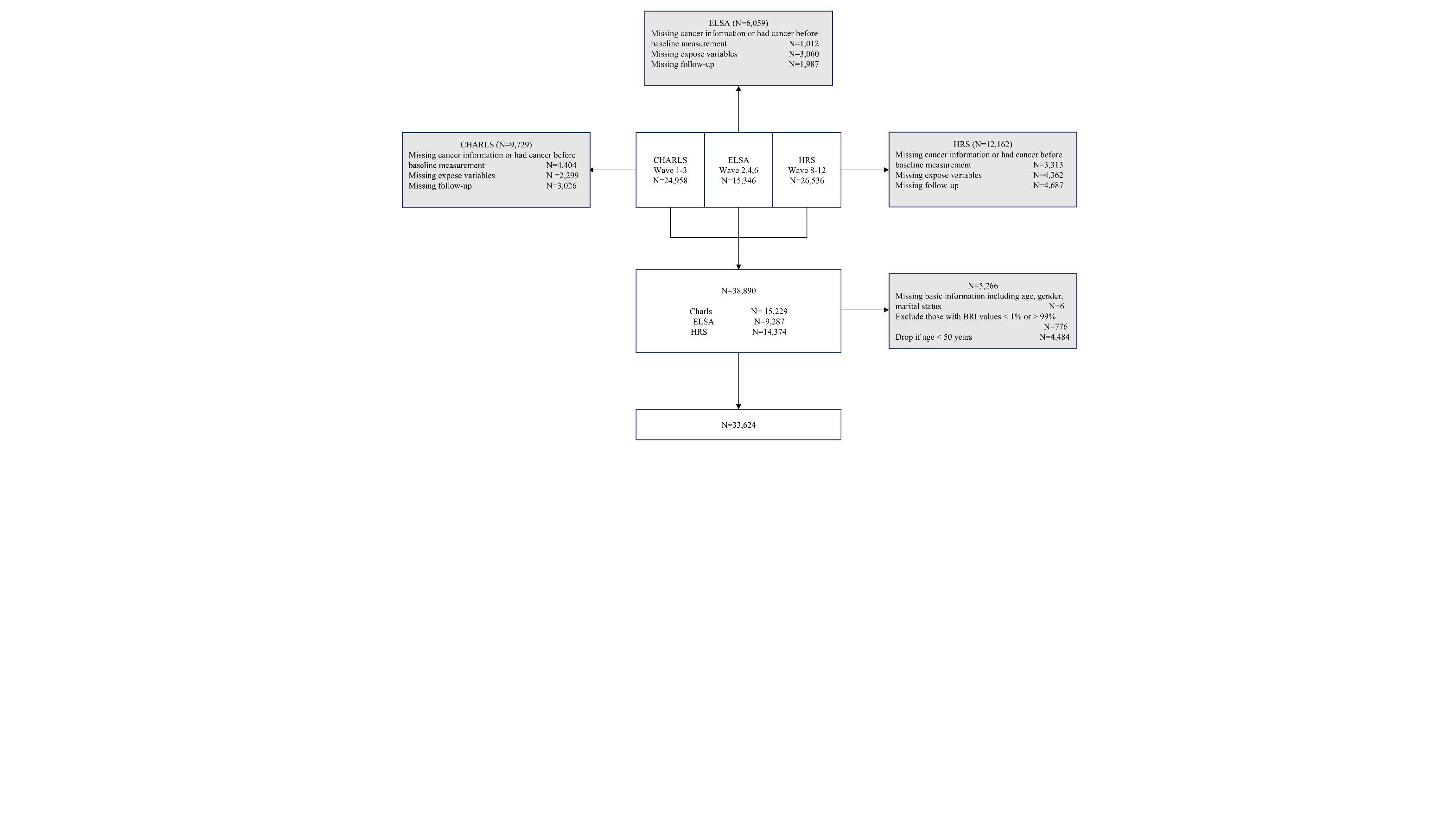


**Figure S2.** Flowchart of data screening from the three cohort studies. **Abbreviation:** ELSA, the English Longitudinal Study of Ageing; HRS, the Health and Retirement Study; CHARLS, the China Health and Retirement Longitudinal Study.


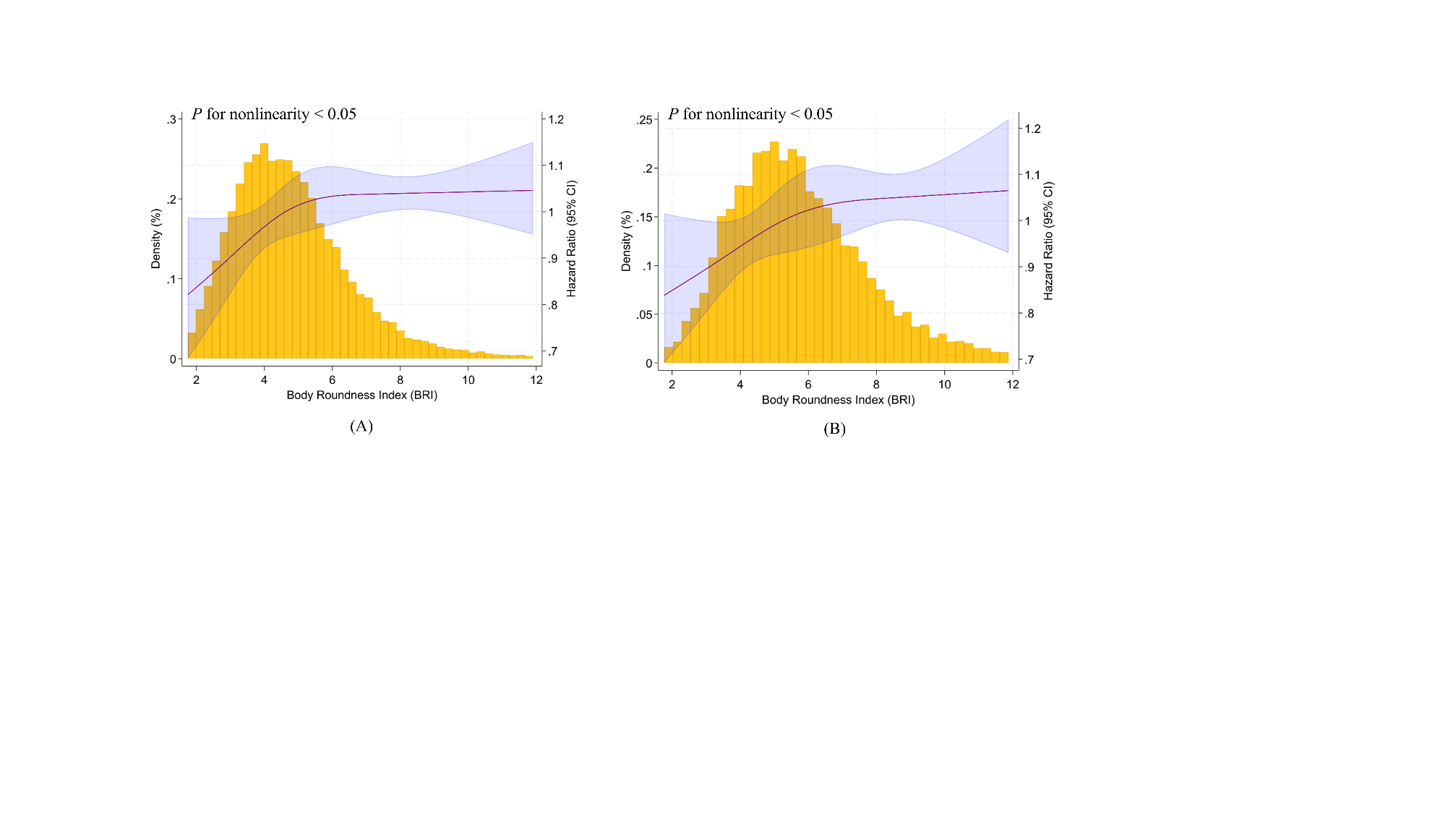


**Figure S3.** Fully adjusted restricted cubic spline curves showing the association between BRI and cancer incidence, stratified by cardiometabolic disease status. (A) Individuals without CMD; (B) Individuals with CMD. *P* for nonlinearity < 0.05 in both groups. Abbreviation: CMD, cardiometabolic disease.


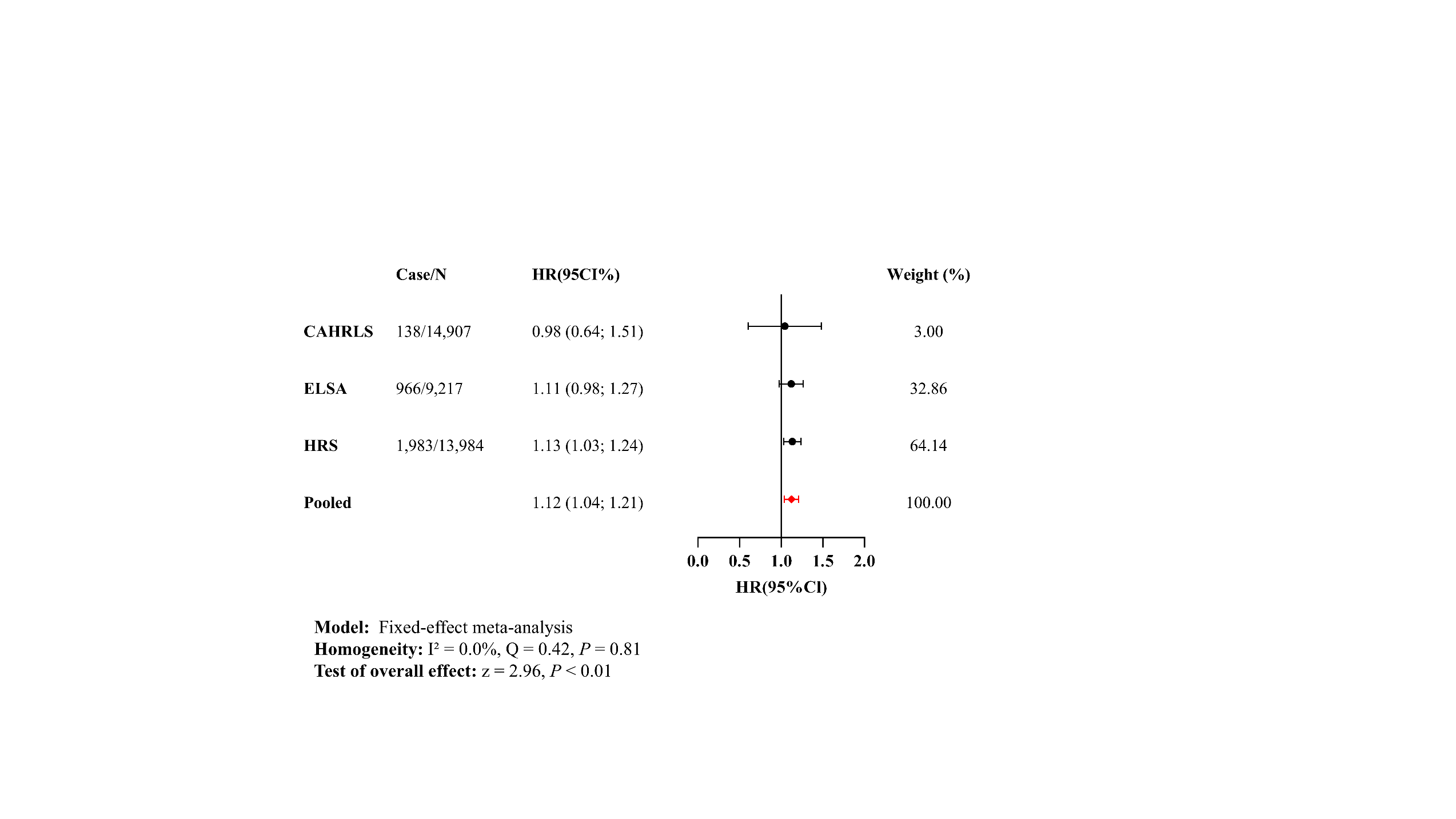


**Figure S4.** Sensitivity analysis synthesizing results from three cohorts via meta-analysis. **Abbreviation:** ELSA, the English Longitudinal Study of Ageing; HRS, the Health and Retirement Study; CHARLS, the China Health and Retirement Longitudinal Study.


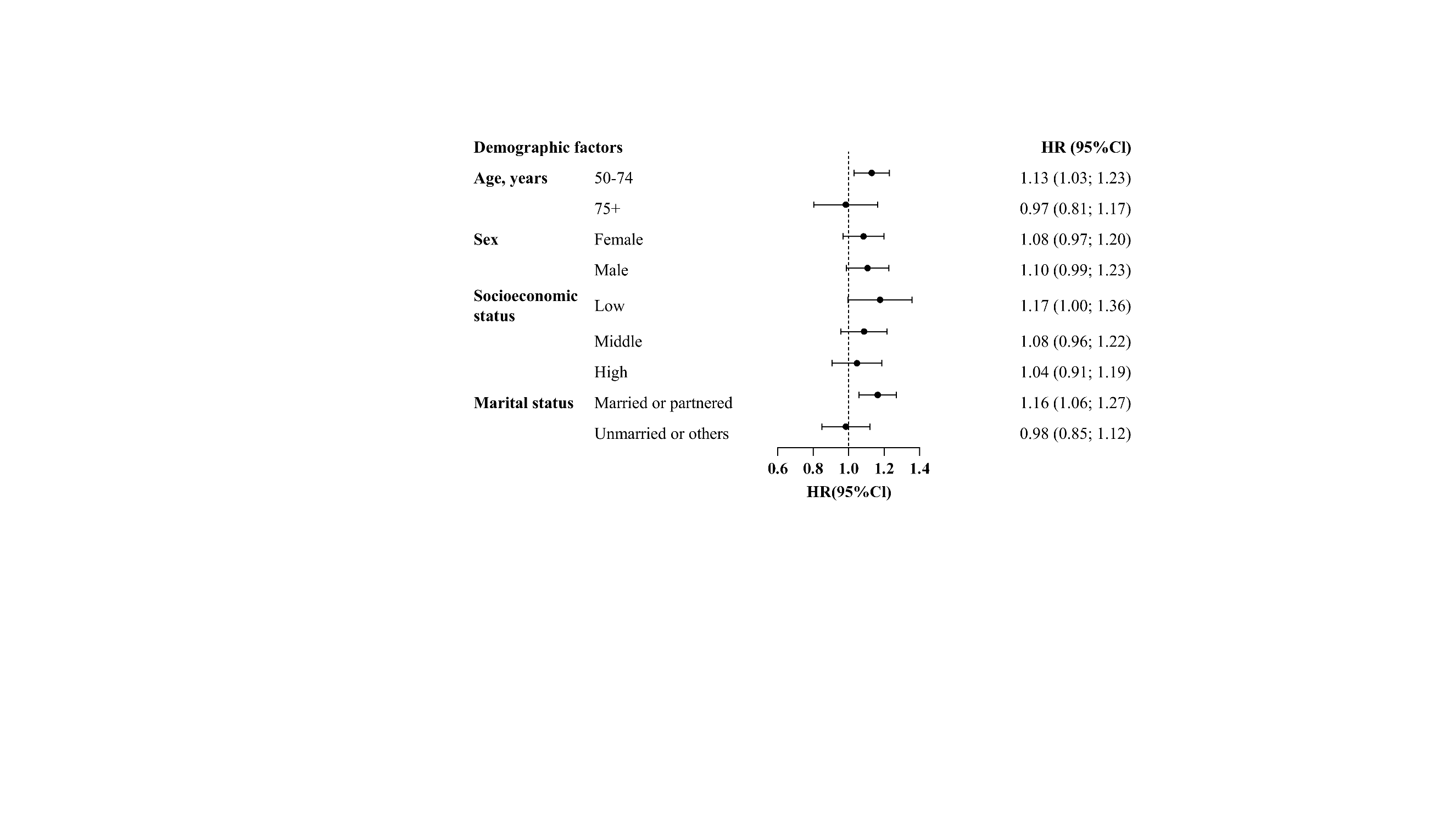


**Figure S5.** The association between BRI and cancer incidence across stratified subgroups. Abbreviation: HRs, hazard ratios; CIs, confidence intervals.


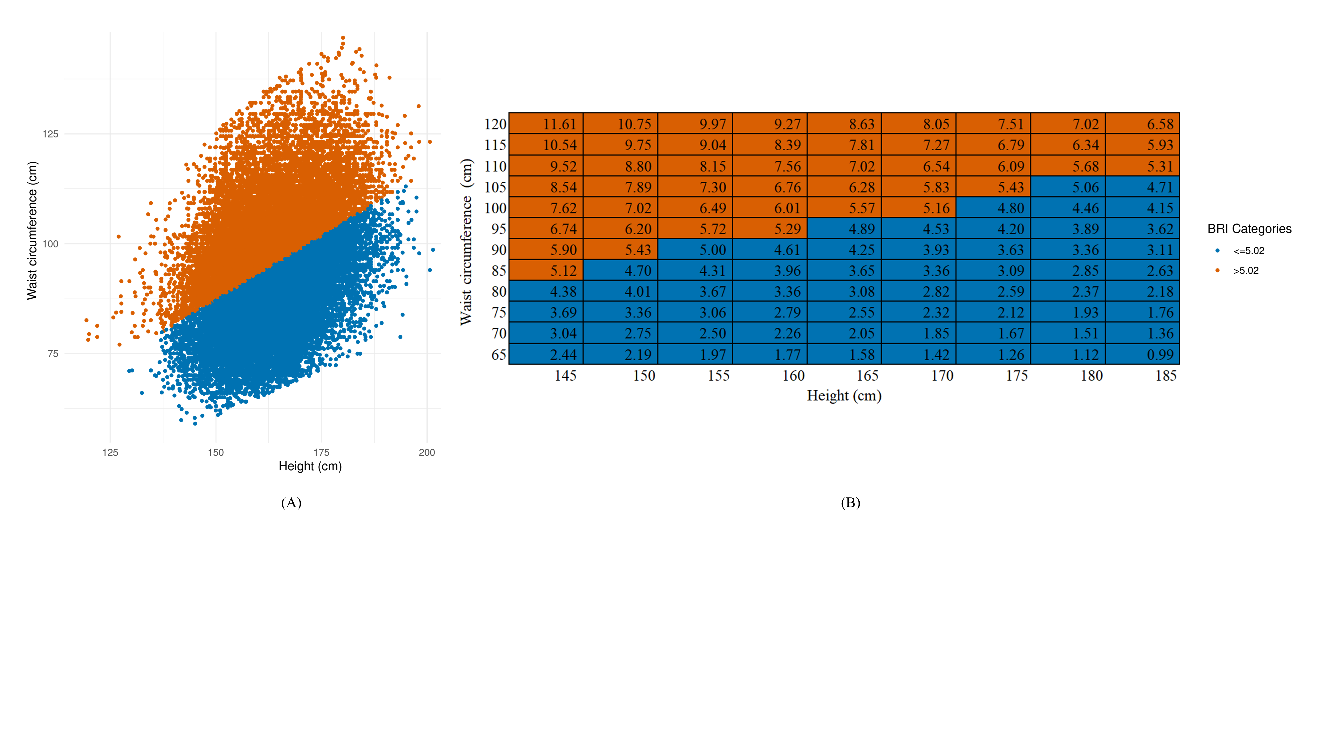


**Figure S6**. BRI categories based on the inflection point and the distribution of height and waist circumferences among individuals aged 50 years and older. (A) Actual distribution of height and waist circumference with BRI categories observed in the study population; (B) A clinical reference grid illustrating BRI categories across height and waist circumference in relation to cancer risk. **Abbreviation:** BRI, body roundness index.
